# Sample Pooling and Inflammation Linked to the False Selection of Biomarkers for Neurodegenerative Diseases in Top–Down Proteomics: A Pilot Study

**DOI:** 10.3389/fnmol.2018.00477

**Published:** 2018-12-18

**Authors:** Nicolas Molinari, Stéphane Roche, Katell Peoc’h, Laurent Tiers, Martial Séveno, Christophe Hirtz, Sylvain Lehmann

**Affiliations:** ^1^Department of Statistics, CHU de Montpellier, University of Montpellier, Montpellier, France; ^2^INSERM, UMR 1251, Aix-Marseille Université, Marseille, France; ^3^APHP, HUPNVS, Hôpital Beaujon, UFR de Médecine Xavier Bichat, Clichy and Université Paris Diderot, Paris, France; ^4^Laboratoire et Plateforme de Biochimie Protéomique Clinique, CHU de Montpellier, Montpellier, France; ^5^CNRS, INSERM, BioCampus Montpellier, University of Montpellier, Montpellier, France; ^6^IRMB, INSERM U1183, University of Montpellier, Montpellier, France

**Keywords:** sample pooling, clinical proteomics, neurodegenerative disease, top–down, serum, CRP, SAA

## Abstract

Proteomic technologies have been recently adapted to the new field of clinical proteomics. The origin of errors and biases has been well-identified in the pre-analytical steps, leading to the measurement of clinical analytes. One possible source of inadequacy in clinical proteomics is linked to sample pooling. This practice is usually related to low sample availability, variability, experiment time/cost. In this study, we first asked whether sample pooling in top–down proteomics is suitable to obtain a relevant biological average. Our second objective was to identify inflammatory biomarkers of outlier samples in our population of Creutzfeldt-Jakob disease patients. Our results demonstrated that, in a proteomics study, sample pooling as well as the inflammation status was an important source of errors: missed detection of biomarkers and false identification of others. Pooled samples were not equivalent to the average of biological values. In addition, this procedure reduced the statistical value of the identified biomarkers due to a stabilization of their standard deviation and rendered outlier samples difficult to detect. We identified serum amyloid A as a candidate biomarker of outlier samples. The presence of this protein, which could be explained by inflammatory processes, induced major modifications in the sample profiles.

## Introduction

Clinical proteomics is a new and expanding domain. Proteomic profiling for discriminating disease states requires high sample numbers and high-throughput capacity. Various proteomic strategies have been developed for discovering new potential biomarkers, and their sensitivity and resolution for detecting peptides, proteins and trypsin-generated peptides are constantly improving. Each technology has its own limitations and advantages. Top–down proteomic approaches focus on the analysis of intact proteins and protein fragments, whereas bottom-up technologies are focusing on peptides resulting from the proteolytic digestion of proteins and peptides. In bottom–up proteomics, the potential biomarkers are immediately identified. Conversely, in top–down proteomics, the complexity of the data requires many purification steps and/or *de novo* protein identification algorithms, limiting the range of protein identification and coverage. SELDI-TOF is one of the top–down approaches initially developed. It can rapidly handle many samples, like MALDI-TOF (200 or more). Conversely, LC-MS-MS has a lower capacity. Regardless of the used technology, the number of samples, the protein amount, and the quality of the pre-analytical steps are essential features. Indeed, inadequate sample quality will affect the fractionation steps (e.g., protein depletion) (Roche et al., 2006; Patel et al., 2012) that allow the investigation of proteins present at low concentration, and also the mass spectrometry analysis. Importantly, the depletion of major proteins might help to detect low abundant proteins, but might also mask some biomarkers.

Besides sample quality, which can be significantly improved by implementing quality control procedures, patient or sample phenotyping also is important, particularly for biological fluids. Blood is a means of communication between organs via growth factors, hormones, and nutrients. Blood composition is influenced by the disease under study, and also by any other unrelated pathology that could affect a patient, such as diabetes (Khan and Awan, 2012) and cardiovascular diseases (Gilstrap and Wang, 2012). This is particularly true in neurodegenerative diseases in which aging also must be taken into account. These unrelated pathologies are generally treated and/or stabilized, and consequently, they are not always recorded by the physician during clinical data collection. However, these unrelated diseases could modify the proteomic profiles, thus decreasing the value of these analyses. In this context, the use of additional clinical biomarkers (of inflammation, renal, metabolic or cardiovascular diseases…) could decrease the risk of outlier profiles due to unrelated diseases. The design of clinical proteomic studies must take into account all these issues to reduce their current variability.

Sample pooling is sometimes used in proteomic studies, and this also could be a major source of artifacts. From a statistical point of view, pooling samples might decrease the study power and modify the mean value or standard deviation of an analyte. From a technological point of view, the effect is more complex. Theoretically, sample pooling is acceptable if the pool represents the biological average of the individual samples. This has been tested and is successfully used in DNA microarray analyses (Zhang and Gant, 2005; Kainkaryam et al., 2012). In proteomics, sample variability seems to be a key point when assessing the suitability of pooling. Therefore, in this study, we analyzed serum samples from patients with Creutzfeldt-Jakob disease (CJD) and controls by top–down proteomics to identify CJD biomarkers. First, we compared the results obtained with individual and pooled samples to determine whether sample pooling represents the biological average in top–down proteomics. Then, we focused on the outlier profiles to identify candidate biomarkers that could be used to detect and eliminate such samples.

## Materials and Methods

### Serum Samples

**FIGURE 1:**
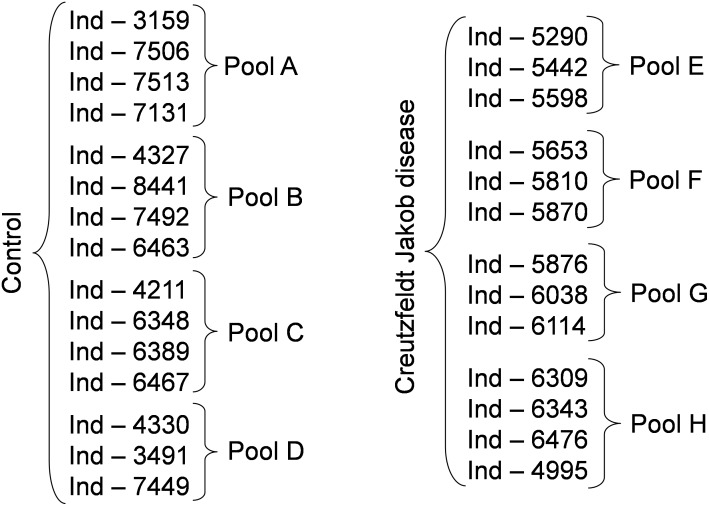
Experimental groups used in the study. To reduce the number of samples to be analyzed we generated pools of 3 to 4 samples, as indicated. Following the identification of possible biomarkers, we analyzed also the individual samples. Ind-number, anonymized number of each individual sample.

Anonymized serum samples were provided by the Lariboisière Hospital Biochemistry and Molecular Biology Department, Paris, France. All investigations were conducted according to the Declaration of Helsinki principles and all participants provided their written informed consent which was a prerequisite for the inclusion in this study. When patients were unable to give their consent, their relatives signed the informed consent. The other inclusion criteria were represented by an age > 18 years old and a CJD diagnosis confirmed by the multidisciplinary team of the Lariboisière Hospital based notably on the 14-3-3 detection in the CSF (Peoc’h et al., 2006). The exclusion criteria were represented by the presence of a neurodegenerative disease other than CJD, and the presence of hemorrhagic CSF. The biological collection was officially registered under the number DRC-2009-953. Blood was collected in vacutainer tubes without additives, left to clot for 30 min at room temperature, and then centrifuged at 3000 ×*g* for 30 min. Serum was recovered and frozen at -80°C until use. For this study, serum samples from two groups (controls without neurodegenerative diseases and from patients with CJD) were assessed. Each sample was analyzed individually and after pooling. Three to four individual samples from the same group were pooled to constitute a pool (Figure 1).

### SELDI-TOF Analysis

Each serum sample was diluted 1.5 times with a solution of 8 M urea, 1% CHAPS and stirred at room temperature for 15 min. ProteinChip Q10 Arrays (anion exchanger) (Bio-Rad) were pre-equilibrated with 150 μl of binding buffer (100 mM Tris pH 9 (made from TrisBase adjusted using HCl solution) and 0.1% Triton X-100) in a 96-well bioprocessor with gentle agitation for 5 min. Then, 2 μl of denaturated sample was mixed with 100 μl of binding buffer. After removing the pre-equilibrated buffer from the wells, denaturated samples were added and incubated on a plate shaker at room temperature for 1 h. Wells were washed twice with binding buffer for 5 min, once with binding buffer without Triton X-100 for 5 min, and finally briefly rinsed with water. The ProteinChip arrays were removed from the bioprocessor and air-dried. Finally, 0.8 μl of saturated sinapinic acid solution was added twice to each spot and arrays were allowed to air-dry.

SELDI-TOF mass spectrometric analysis was performed on a PBS-II SELDI ProteinChip reader using the following settings for all samples and for data collection: laser intensity 270, detector sensitivity 8, molecular mass range 2,000 to 20,000 m/z, center mass 12,500 m/z, 80 shots per spot. The ProteinChip All-in-One Protein Standard II (Bio-Rad) was used for external calibration. Spectrum analysis was performed using the ProteinChip software version 3.2 (Bio-Rad). The background was subtracted using the default software settings. Peaks with a signal/noise ratio above three were identified by the ProteinChip software (see Supplementary Table S1). After normalization to the total ion current (TIC) and quantification, data were exported to R version 2.1.1, for statistical analyses.

### Statistical Analysis

Using the peaks identified by the ProteinChip software, peaks with signal intensities that were significantly different between patients and controls were detected using the Student’s *t*-test for normally distributed, and the non-parametric Mann–Whitney *U*-test for non-normally distributed peak intensities. The Shapiro–Wilk test was used to assess the normality assumption. A Bonferroni correction was applied to take into account the large number of repeated tests.

To compute the smoothing index between individual samples and their pool, the variation between the individual peak intensities and the corresponding pool peak intensity was calculated. The smoothing index was computed using the following formula: index(m/z) = 1/(n-1) Σ_individual_ (individual(m/z)-pool(m/z))^2^, where the standard deviation formula was adapted by considering the pooled value as a classical mean value. This index represents the local smoothing of the pool for the peaks at m/z. A large index indicates a large smoothing between individual and pooled values.

### Protein Identification

A volume of 50 μL of each serum sample was diluted 1.5 times with a solution of 8 M urea, 1% CHAPS and stirred at room temperature for 15 min. After addition of 2 mL of 100 mM Tris pH9 (corresponding to TrisBase adjusted using HCl solution) and 0.1% Triton X-100, samples were agitated in ProteinChip Q Spin Columns (Bio-Rad) at 4°C for 2 h. After three washes in 100 mM Tris pH 9/0.1% Triton X-100, two samples were eluted with 150 μL of 100 mM Tris pH8/0.1% Triton X-100. Samples were concentrated and separated on 12% NuPage gels (Invitrogen) with MES buffer (Invitrogen) as running buffer. Gels were then dehydrated in 50% ethanol/10% acetic acid, rinsed in 50% ethanol once, and in water three times, and then stained with colloidal Coomassie blue (PageBlue Protein Staining Solution, Fermentas).

Protein bands were excised from the gels and washed in 15 μl of 100 mM NH_4_HCO_3_ for 10 min. After incubation with 15 μl of acetonitrile for 10 min, supernatants were removed and the procedure repeated. After vacuum drying in a SpeedVac apparatus, protein bands were re-hydrated in 10 μl of trypsin solution (15 ng/μl, Promega) and digested in 10 μl of 100 mM NH_4_HCO_3_/5 mM CaCl_2_ buffer at 25°C overnight. Digested peptides were extracted using a two-step procedure. First, 10 μl of 100 mM NH_4_HCO_3_ was added, followed by 10 μl of acetonitrile left for 10 min. This step was repeated twice and supernatants pooled. Second, samples were incubated with 10 μl of 5% formic acid for 10 min, and then 10 μl of acetonitrile was added for 10 min. This step was repeated twice, and the two supernatants were pooled. After complete drying, pellets were resuspended in 10% formic acid.

Each sample (1 μL) was analyzed online using Nanoflow HPLC-Nanoelectrospray Ionization on a quadrupole time-of-flight mass spectrometer (QSTAR Pulsar-I, Applied Biosystems, Foster City, CA, United States) coupled to an Ultimate 3000 HPLC (Dionex, Amsterdam, Netherlands). Sample desalting and pre-concentration were performed online on a PepMap^®^ precolumn (0.3 mm × 10 mm, Dionex). A gradient consisting of 0–40% B for 30 min, 40–80% B for 15 min (A = 0.1% formic acid, 2% acetonitrile in water; B = 0.1% formic acid in acetonitrile) at 300 nL/min was used to elute peptides from a PepMap^®^ capillary (0.075 mm × 150 mm) reversed-phase column (Dionex).

Spectra were recorded using the Analyst QS 1.1 software (Applied Biosystems). All MS/MS spectra were searched against *Homo sapiens* entries of the Swiss-Prot and TrEMBL databases (Sprot_Trembl_20100301), using the Mascot V 2.2 algorithm^1^ and the following parameters: peptide mass tolerance of ±0.2 Da, fragment mass tolerance of ±0.2 Da, methionine oxidation as variable modification, and one trypsin missed cleavage allowed. Peptides with scores higher than the identity score (*p* < 0.05) were considered as significant.

### C-Reactive Protein Quantification

C-reactive protein (CRP) in serum samples was quantified with the CRP immunoturbidimetric kit (Randox) and an Olympus AU 640 Chemistry Analyzer (Olympus, Rungis, France) (Dupuy et al., 2007).

## Results

### Pools and Biomarker Discovery

**FIGURE 2:**
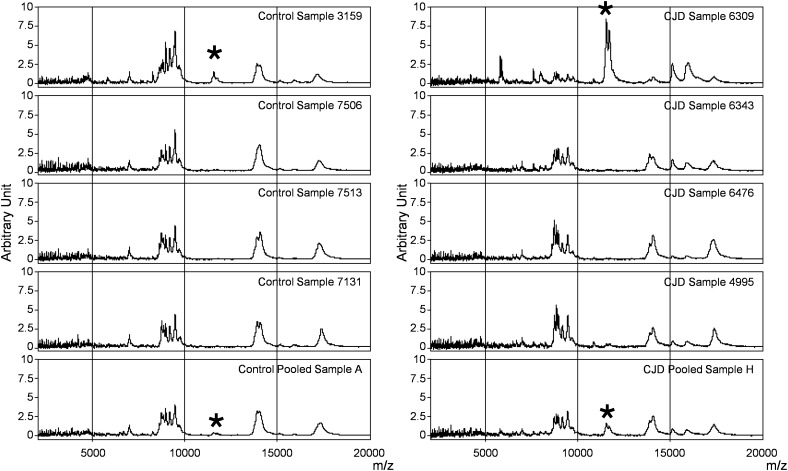
SELDI-TOF profiles of individual and pooled samples in the control and CJD groups. Representative profiles of the mass spectrometry profiles obtained for each group. In each column, the first four profiles belong to four individual serum samples, and the last one is from the pool composed by these individual samples. The asterisk shows the peaks at m/z 11 514, 11 672, and 11 736 identified as SAA.

**FIGURE 3:**
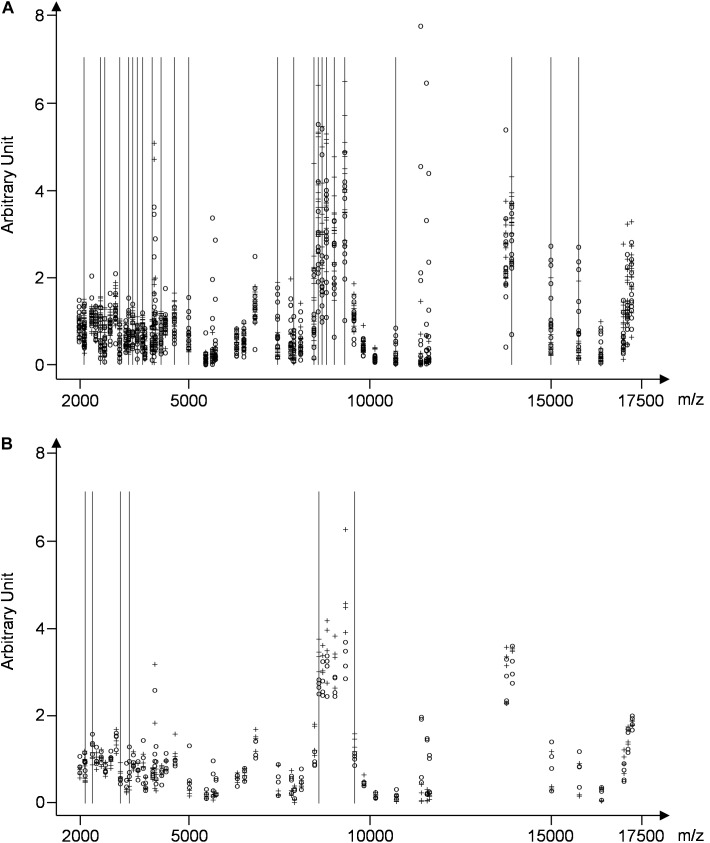
Diagrams showing the peak intensities obtained by SELDI-TOF analysis of individual **(A)**, and pooled samples **(B)** from the control group (**○**) and the CJD group (**+**) (see Supplementary Table S2). The vertical lines indicate peaks with different intensities between controls and patients with CJD.

**FIGURE 4:**
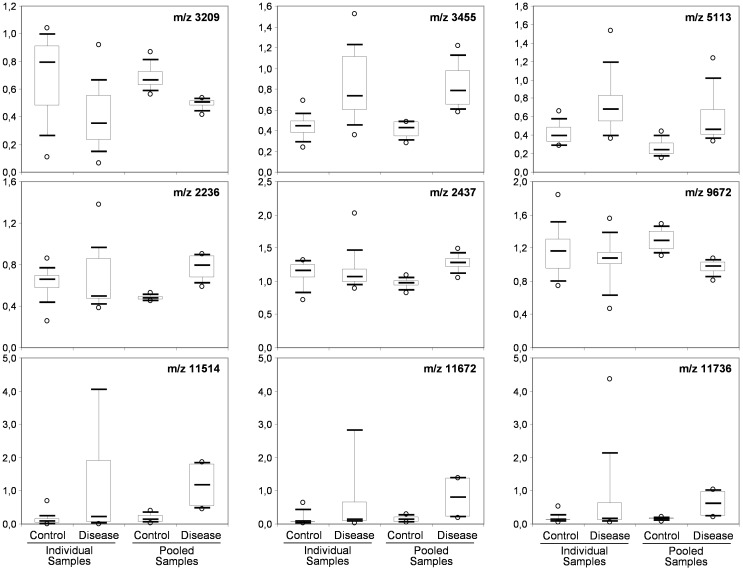
Box-and-whisker diagrams of differential peaks. The first three peaks **(top)** were identified as having different intensities in the control and CJD groups (not true for 5113) the individual and the pooled samples analyses. The second three peaks **(middle)** were identified as differential peaks only in the pooled sample analysis. The last tree peaks **(bottom)** represent the proteomic signature of outlier samples (SAA peaks).

To determine whether sample pooling resulted in some unforeseen methodological and statistical bias, or performed like a biological average, individual or pooled serum samples from controls and patients with CJD (Figure 1) were analyzed by SELDI-TOF on Q10 anion-exchange ProteinChip Arrays. Pooling reduced the number of samples to analyze from 15 to 4 for the control group, and from 13 to 4 for the CJD group. The m/z ratio ranged from 2,000 to 20,000 (Figure 2), and 54 clusters could be detected (see Supplementary Table S3). Compared with the individual sample analysis, sample pooling affected the coefficient of variation minimal, maximal, and mean values in both control and CJD groups (Table 1). In individual samples, 24 peaks (m/z 2224, 2679, 2795, 3209, 3455, 3567, 3694, 3840, 4104, 4349, 4718, 5113, 7565, 8012, 8568, 8686, 8792, 8911, 9128, 9419, 10824, 14024, 15106, and 15872) were identified as having a different intensity between controls and the CDJ group (Figure 3A). As an example, the intensities of the peaks at m/z 3209, 3455, and 5113 were plotted in a box-and-whisker diagram (Figure 4). Sample pooling reduced the number of differential peaks to six (m/z 2236, 2437, 3209, 3455, 8686, and 9672) (Figure 3B), among which three peaks (m/z 2236, 2437, and 9672) were not identified in the individual sample analysis (Figure 4). Compared with individual samples, pooling influenced the standard deviation of the potential biomarkers, and decreased peak variability (Figure 4). Consequently, the value of statistical tests that depends on standard deviation was also affected.

**Table 1:** Statistical analysis of SELDI-TOF mass spectra.

	Control		Neurodegenerative disease	
	Individual	Pooled	Individual	Pooled
Sample number	15	4	13	4
Mean CV	60.9%	44.3%	69.4%	35.7%
Minimal CV	13.9%	1.0%	26.2%	5.2%
Maximal CV	181.3%	124.0%	193.1%	85.7%

*The minimal, maximal, and mean coefficient of variation (CV) values for each group (Control and Neurodegenerative Disease, Individual and Pooled samples) were calculated using each peak of each profile.*

### Sample Pooling and Biological Average

**FIGURE 5:**
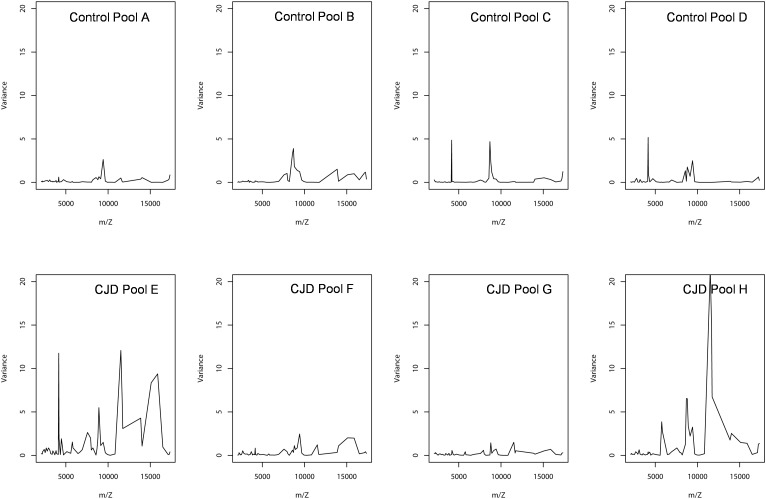
Peak intensity variance between individual and pooled samples. The pooled sample values were used as the biological average of the individual values. The variance was computed using a standard deviation formula, with the pooled intensity as the average.

A smoothing index was used to compute the variation between the individual sample values and the biological average (pooled samples) for all peaks in all spectra. The formula of standard deviation was adapted to compute this index. The smoothing index was not homogenous for all peaks within a pool (Figure 5). For instance, in pool E (CDJ group), some peaks had a very high smoothing index (higher than 10). This observation also applied to the other profiles, but with lower values. Moreover, 10 peaks (18.5%) in the control group showed a variation above 20% between pooled and individual samples. The number of peaks increased to 30 (55%), when this variation was set at 10%. In the CJD group, 13 peaks (24%) displayed a variation of 20%, and 37 peaks (68.5%) a 10% variation.

### Outlier Biomarker Identification

**FIGURE 6:**
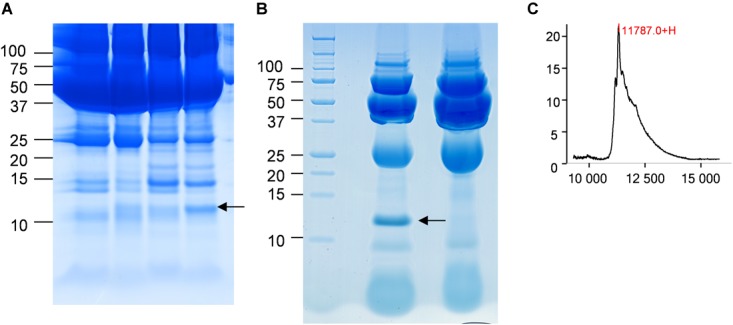
Identification of the candidate biomarker in outliers. **(A)** Four CJD samples with a 10–15 kDa band of different intensity (arrow). **(B)** Electrophoresis analysis of the purified candidate biomarker. The arrow marks the position of the peak. **(C)** After passive elution of the band in **(B)**, the presence of the candidate biomarker was confirmed by SELDI-TOF analysis with a Q10 ProteinChip Array.

**FIGURE 7:**
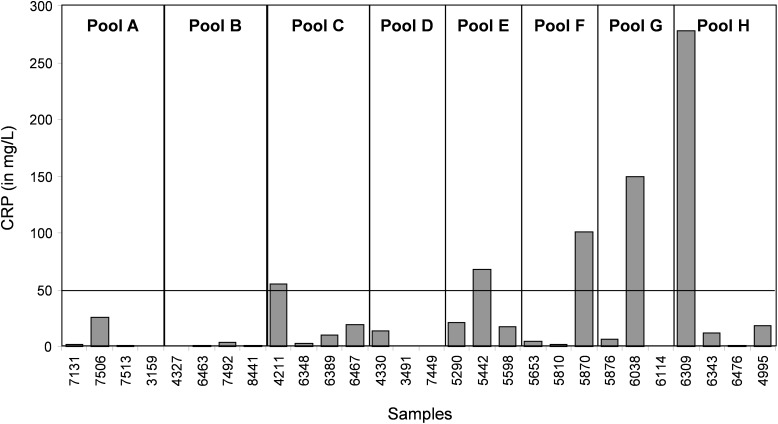
Quantification of CRP (a biomarker of inflammation and infectious diseases) in each individual serum sample.

In the CJD group, a strong increase of the smoothing index was observed (Figure 5). A peak at m/z 11,514 was observed in few individual CJD samples and in one sample per pool (Figure 2, the peak marked with a star). This peak was related to the high value of the smoothing index in pools E and H (12.08 and 21.36, respectively) (Figure 5), and was correlated with two other peaks at m/z 11,672 and 11,736 (0.983 and 0.991, respectively). These two other peaks had deviation/smoothing indices of 6.32 and 3.09 (pool E) and 14.95 and 6.73 (pool H). The coefficients of variation for these three peaks were 181, 145, and 79% for the control group, and 169, 193, and 164% for the CJD group, respectively. In pooled samples, this variability was reduced to 95, 81, and 36% for the control group, and to 66, 86, and 71% for the CJD group (Figure 4, m/z 11,514, 11,672, and 11,736). These peaks were correlated with two other peaks at m/z 5,768 and 5,848. Their mass peaks were two times lower than those of the first three peaks and might be the molecular form MH^2+^. To identify the first three peaks, biochemical purification was performed (see section “Materials and Methods”), and at each step, the presence of the protein of interest was checked using SELDI-TOF mass spectrometry. Finally, after SDS-PAGE electrophoresis, a band with a molecular weight between 10 and 15 kDa (Figures 6A,B) was detected and the SELDI-TOF spectra indicated that it was composed mainly of a protein with an m/z of 11,787 (Figure 6C). After *in gel* trypsin digestion and LC-MS-MS analysis, comparison of the mass values in the SwissProt database identified seven serum amyloid A (SAA) peptides (access number in SwissProt: SAA_HUMAN) (Supplementary Figure S1), and also four peptides from four different proteins (Supplementary Table S3). More sensitive proteomics approaches, such as the high resolution Q-TOF technology (Vialaret et al., 2018), could have allowed the identification of more proteins in this band. SAA is an acute-phase inflammatory effector (Sun and Ye, 2016), and is often detected in proteomic studies (Cho et al., 2004; Bozinovski et al., 2008; Brea et al., 2009; Findeisen et al., 2009; Meling et al., 2013). The presence of this cluster of three peaks has already been described in SELDI-TOF analyses of samples from patients with various pathologies (Tolson et al., 2004; Findeisen et al., 2009). To confirm this result, the correlation between SAA and CRP level was assessed by quantifying CRP (Figure 7) in each serum sample, and then by comparing this value with the presence of the three peaks at m/z 11,514, 11,672, and 11,736. The correlation factors were 0.91, 0.87, and 0.91 for each peak, respectively. Moreover, a CRP cut-off value of 50 mg/L could detect the outlier samples with good specificity (100%) and sensitivity (96.1%).

## Discussion

### Pooling Samples Might Lead to False Biomarker Discovery

The decision of pooling samples is a delicate issue due to its impact on the study design (Karp and Lilley, 2007; Oberg and Vitek, 2009). It was previously shown that sample pooling has an influence on demographic and clinical data (Zhang and Gant, 2005; Oberg and Vitek, 2009; Kainkaryam et al., 2012). In this work, we focused on the impact of sample pooling on proteomics results. By definition, pooling decreases the number of samples analyzed and therefore, modifies the standard deviation of the results (Karp and Lilley, 2009). It also increases the total amount of sample that can be analyzed. This is beneficial because sample amount often represents a limitation to discover new biomarkers in high through-put proteomic analyses using recent technological methods, such as fractionation, chromatography and high-sensitivity mass spectrometry. This is particularly true for biological samples with low protein concentration, such as cerebrospinal fluid in which protein amount is usually lower than 0.4 g/L and reaches a maximum of 5 g/L (in infectious diseases) (Roche et al., 2008; Gabelle et al., 2009). However, sample pooling does not completely solve the problem of sample shortage because pooling is often compensated by increasing the number of replicates (Karp and Lilley, 2009). The main problem in clinical proteomics remains to have a number of samples high enough to reach statistical significance. Indeed, significance is based on statistical tests related to the value of the standard deviation that needs to be reduced to increase the probability of discovering a new biomarker. This can be obtained by increasing the sample number, by standardization of the pre-analytical steps, and/or by pooling samples. Our results actually demonstrated that sample pooling decreases the power of statistical tests (e.g., the probability that the test will reject a false hypothesis of equivalent groups), and results in a lower number of differential peaks. In our experiment, 24 differential peaks were identified in individual samples, and only six in pooled samples (Figure 3). In parallel, sample pooling increased the detection of false differential peaks (m/z 2236, 2437, and 9672 in Figure 4), due to a reduction of their standard deviation. This explains why some false positive peaks appear in pooled studies (Sadiq and Agranoff, 2008; Colegrave and Ruxton, 2017). Taken together, our observations are in agreement with previous studies (Sadiq and Agranoff, 2008; Diz et al., 2009; Karp and Lilley, 2009).

### Biological and Mathematical Averaging Are Not the Same in Proteomics

Sample pooling is suitable for proteomic analyses only when they are representative of the individual samples used to constitute the pool. Mathematically, this has been defined as the Jensen’s inequality (Jensen, 1906): if the pool value is equal to the average value of the individual samples used to make it, the assumption of biological averaging holds, and sample pooling is possible and beneficial. To test the assumption of biological average, we analyzed the variations between individual and pooled sample results using a smoothing index, adapted from the formula of the standard deviation (Figure 5). In pools E and H, the higher smoothing index indicated that the pools were different from the included individual samples. In the other pools, the smoothing index was lower than in pools E and H for all peaks. This led us to conclude that the assumption of biological averaging does not hold for protein profiles. This might be due to protein-protein interactions during the pre-analytical and analytical steps (Sadiq and Agranoff, 2008), or to the homogeneity of the patient population used. Karp and colleagues have proposed that for a high degree of biological variation, the Jensen’s inequality becomes significant, and the assumption of a biological averaging does not hold. They also suggested that human samples are more variable than mouse samples (Karp and Lilley, 2009). Here, we confirmed that for human samples with high standard deviation, the assumption of biological averaging does not hold. Using the outlier samples, we propose a mechanism to explain this discrepancy.

### Identification of an Inflammation Biomarker to Detect Sample Outliers

In our study, we used samples from a control group without neurodegenerative diseases and a group of patients with CJD, a pathology characterized by progressive dementia and fatal outcome in 4 to 20 months in most sporadic cases (Ironside et al., 2017). Our proteomic analysis highlighted the presence of few individual profiles that were not comparable to the others. A biomarker to identify and remove these outliers before proteomic analysis would greatly increase the significance of the analysis. Here, we found that SAA is such a candidate. This protein is considered a biomarker of various pathologies, including prion diseases (Meling et al., 2013). CRP is a validated inflammation biomarker that is widely used for inflammatory disease diagnosis. Both CRP and SAA are produced in response to similar cytokine and pro-inflammatory stimuli (Steel et al., 1996; O’Hara et al., 2004).We found that the outliers were from patients with a CRP value above 50 mg/L or with an SAA value above 0.5 AU. These outliers induced artifacts when samples were pooled, and were not comparable with other spectra at the individual level.

## Conclusion

We have demonstrated that sample pooling in our top–down proteomic approach does not represent the biological average of the individual samples. This finding is critical for proteomic studies of biological fluids due to the complexity of the samples, pre-analytical steps and technologies. We found that in some samples, inflammatory-related factors can result in specific profiles not related to the neurodegenerative disease. These outlier samples might be identified using SAA and CRP as possible biomarkers. We think that sample pooling remains an option if certain rules are taken into account, such as sample homogeneity and increasing the number of replicates to maintain the statistical significance. However, for human samples, the complexity of the pathologies under study and the potential interference from unrelated diseases greatly limit the interest of sample pooling.

## Ethics Statement

The study was authorized by the ethical committee CPP Sud Méditerranée IV under the number 08 03 06 and by the Health Authorities under the number DGS2008-0076.

## Author Contributions

SL, NM, and SR designed the study. SR, LT, and MS performed experiments and analyzed data. SL, NM, KP, SR, and CH interpreted the results. SL, NM, SR, and CH wrote the manuscript draft. All authors critically revised the manuscript and approved its contents before submission.

## Conflict of Interest Statement

The authors declare that the research was conducted in the absence of any commercial or financial relationships that could be construed as a potential conflict of interest.

## Footnotes

1 http://www.matrixscience.com

## Supplementary Material

FIGURE S1 Identification of the candidate protein by *in gel* trypsin digestion and LC-MS-MS. The mass values were compared with those in the SwissProt database and seven peptides were identified as belonging to “Serum Amyloid Protein A” (Access number in SwissProt: SAA_HUMAN). **(a–g)** MS/MS fragment spectrum (CID) of the 7 peptides identifying Serum Amyloid Protein A.

TABLE S1 Identification results obtained by LC-MS-MS.

TABLE S2 Peak intensity and signal/noise ratio (SN) of individual and pool samples (see Figure 3).

TABLE S3 Peak intensity of individual and pooled samples from the control and CJD groups (see Figure 2).
