# Supplementary figures and images for: Sample Pooling and Inflammation Linked to the False Selection of Biomarkers for Neurodegenerative Diseases in Top–Down Proteomics: A Pilot Study
Source: Front Mol Neurosci. 2018 Dec 18;11:477. doi: 10.3389/fnmol.2018.00477 (PMC6305369; doi:10.3389/fnmol.2018.00477)

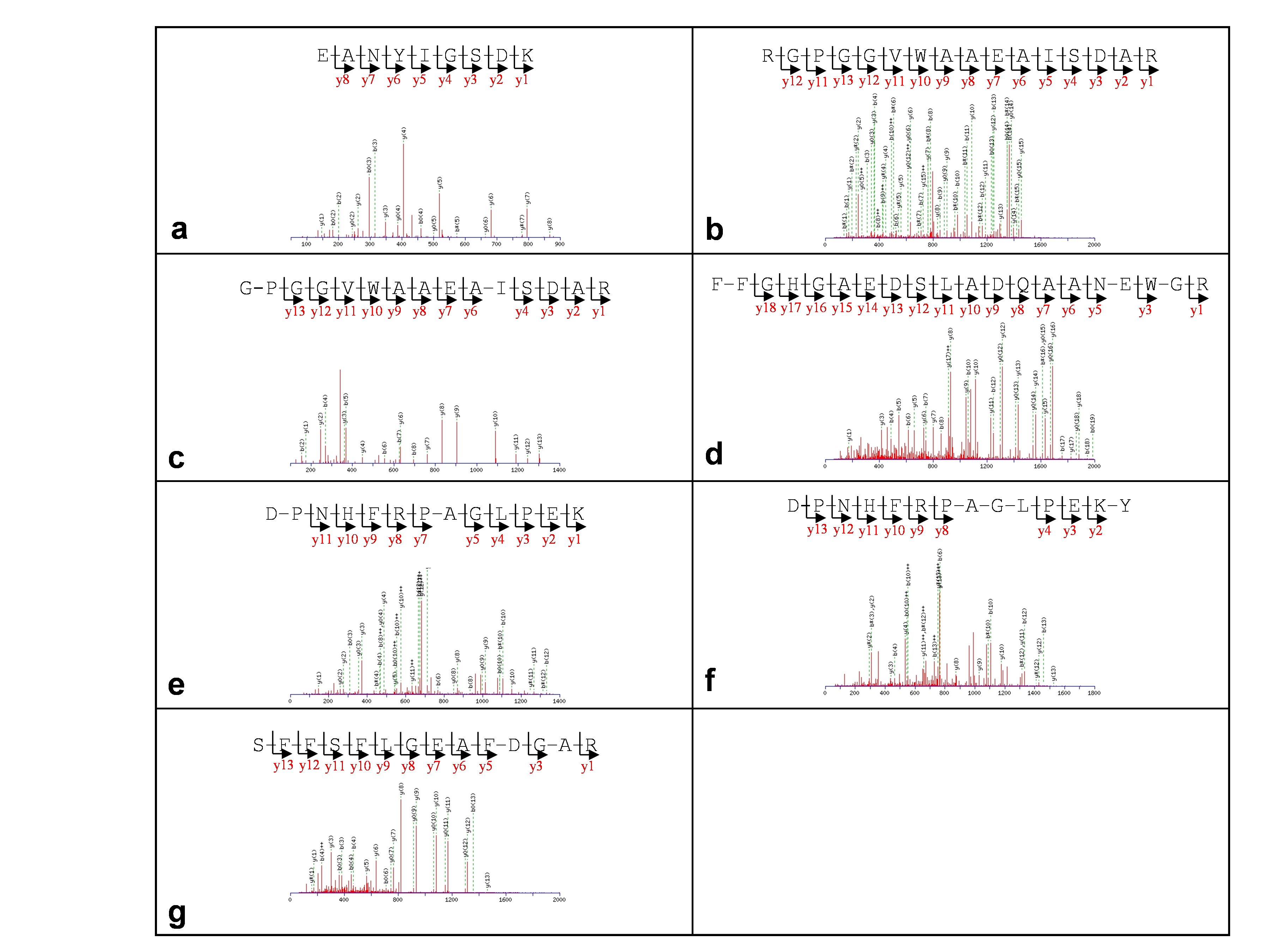

Supplement: FIGURE S1 — Identification of the candidate protein by in gel trypsin digestion and LC-MS-MS. The mass values were compared with those in the SwissProt database and seven peptides were identified as belonging to “Serum Amyloid Protein A” (Access number in SwissProt: SAA_HUMAN). (a–g) MS/MS fragment spectrum (CID) of the 7 peptides identifying Serum Amyloid Protein A. [file Image_1.TIF]
