# Supplementary material for: Sample Pooling and Inflammation Linked to the False Selection of Biomarkers for Neurodegenerative Diseases in Top–Down Proteomics: A Pilot Study
Source: Front Mol Neurosci. 2018 Dec 18;11:477. doi: 10.3389/fnmol.2018.00477 (PMC6305369; doi:10.3389/fnmol.2018.00477)
Supplement: TABLE S1 — Identification results obtained by LC-MS-MS. [file Table_1.DOC]

**Sub Table 1**: Identification results obtained by LC-MS-MS.

| SwissProt Access Number | Protein Name | Theoretical Mass | Peptide Mass | Peptide Score | Peptide Theoretical Sequence |
| --- | --- | --- | --- | --- | --- |
| SAA_HUMAN | Serum amyloid A | 13524 | 7 | 60.1 | EANYIGSDK |
|  |  |  |  | 101.71 | GPGGVWAAEAISDAR |
|  |  |  |  | 48.76 | DPNHFRPAGLPEK |
|  |  |  |  | 84.57 | SFFSFLGEAFDGAR |
|  |  |  |  | 86.74 | RGPGGVWAAEAISDAR |
|  |  |  |  | 36.33 | DPNHFRPAGLPEKY |
|  |  |  |  | 134.22 | FFGHGAEDSLADQAANEWGR |
| KAC_HUMAN | Ig kappa chain C region | 11602 | 1 | 146.04 | VDNALQSGNSQESVTEQDSK |
| APOC3_HUMAN | Apolipoprotein C-III | 10846 | 1 | 114.76 | DALSSVQESQVAQQAR |
| LAC_HUMAN | Ig lambda chain C regions | 11230 | 1 | 75.46 | YAASSYLSLTPEQWK |
| IGHG1_HUMAN | Ig gamma-1 chain C region | 36083 | 1 | 65.59 | TTPPVLDSDGSFFLYSK |
